# The NLRP3 Inflammasome as a Central Driver of Mastitis Pathogenesis: A Review

**DOI:** 10.3390/vetsci13070609

**Published:** 2026-06-24

**Authors:** Shuaishuai Wu, Mohamed Tharwat, Ibrahim F. Halawani, Fuad M. Alzahrani, Khalid J. Alzahrani, Muhammad Zahoor Khan

**Affiliations:** 1 College of Animal Science and Technology, Henan University of Animal Husbandry and Economy, Zhengzhou 450046, China; 2 Department of Clinical Sciences, College of Veterinary Medicine, Qassim University, P.O. Box 6622, Buraidah 51452, Saudi Arabia; atieh@qu.edu.sa; 3 Department of Clinical Laboratory Sciences, College of Applied Medical Sciences, Taif University, P.O. Box 11099, Taif 21944, Saudi Arabiafuadmubarak@tu.edu.sa (F.M.A.);; 4 College of Agriculture and Biology, Liaocheng University, Liaocheng 252000, China

**Keywords:** NLRP3 inflammasome, mastitis, pyroptosis, blood–milk barrier, gut–mammary axis, pharmacological inhibition

## Abstract

Mastitis remains the most economically damaging disease of dairy production, and recent molecular work has converged on the NLRP3 inflammasome as a key integrative node of its pathogenesis. This narrative review integrates evidence published largely between 2015 and 2026 to show how diverse triggers—*Staphylococcus aureus* and *Escherichia coli*, lipopolysaccharide (LPS) and lipoteichoic acid (LTA), non-esterified fatty acids (NEFA), heat stress, environmental xenobiotics including nanoplastics, and microbiota-derived signals—may funnel into a common NLRP3–ASC–caspase-1–GSDMD axis that drives pyroptosis, blood–milk barrier disruption, and clinical disease. The review examines the potential obligatory role of reactive oxygen species (ROS), mitochondrial dysfunction, and selenoprotein-mediated redox control in licensing inflammasome assembly. It further evaluates the emerging gut–mammary and rumen–mammary axes that operate upstream of local epithelial activation. We survey a structurally diverse therapeutic landscape encompassing dietary selenium, probiotics, microbial metabolites, plant-derived nanovesicles, polyphenols, ginsenosides, and small-molecule NLRP3 antagonists, identifying recurring mechanistic motifs that suggest combinatorial regimens may yield additive benefit. Importantly, much of the evidence derives from in vitro and murine models, and we highlight the translational gaps that must be bridged before clinical application in dairy cattle. Finally, we map outstanding research gaps and propose priorities for translational work aimed at sustainable, antibiotic-sparing management of bovine mastitis.

## Simple Summary

Mastitis, an inflammation of the mammary gland, is the most common and economically costly disease in dairy farming worldwide, causing reduced milk production, animal welfare concerns, and increased reliance on antibiotics. Rising antimicrobial resistance has made it urgent to understand the underlying biology of the disease and to identify alternative therapeutic strategies. Recent research has highlighted that a protein complex called the NOD-like receptor family, pyrin domain containing 3 (NLRP3) inflammasome plays a pivotal role in how the mammary gland responds to infection and stress. This review brings together studies showing that bacteria, dietary imbalances, heat, environmental pollutants, and gut microbial disturbances all act through this single inflammatory hub to damage udder tissue. We discuss how nutrition, particularly selenium, gut and rumen microbial balance, and natural plant-based compounds can modulate this pathway, offering safer, antibiotic-sparing options for prevention and treatment. By integrating findings from molecular biology, animal nutrition, and microbiome science, we provide a roadmap for sustainable mastitis management in modern dairy production.

## 1. Introduction

The mammary gland is a dynamic tissue whose lobulo-alveolar architecture, high secretory activity, and frequent exposure to external pathogens through the teat canal make it particularly vulnerable to inflammation [1,2]. In dairy cattle, mastitis remains the most prevalent and economically damaging disease, reducing milk yield, altering milk composition, increasing culling rates, and inflating treatment costs [3]. The disease is multifactorial, involving complex interactions among host, pathogen, environment, and the gut–mammary microbiome axis, with bacterial pathogens such as *Escherichia coli*, *Staphylococcus aureus*, *Streptococcus agalactiae*, and *Klebsiella* spp. being the most frequent etiological agents [4].

Selective breeding for higher milk yield has unintentionally co-selected for increased susceptibility to mastitis [5,6], and the rise in antimicrobial resistance has intensified the search for alternative biological strategies [7,8,9,10]. Although genetic markers associated with mastitis resistance have been identified in immune-related genes, including Toll-like receptors, chemokines, and β-defensins [11,12,13,14,15,16,17,18], the polygenic etiology of mastitis necessitates further validation of candidate markers before routine genomic application.

Among the inflammation-related candidate genes currently under investigation, NOD-like receptor family, pyrin domain containing 3 (NLRP3) appears to be particularly noteworthy in experimental studies and may warrant further investigation in field settings. Unlike the markers described above, which act primarily in pathogen recognition and early immune signaling, NLRP3 encodes a cytosolic pattern-recognition receptor that detects both pathogen-associated molecular patterns (PAMPs) and damage-associated molecular patterns (DAMPs) and orchestrates the assembly of the inflammasome complex [19,20]. Through caspase-1 activation, NLRP3 governs the maturation of IL-1β and IL-18 and the cleavage of gasdermin-D, thereby coupling innate immune sensing to cytokine release and pyroptotic cell death [19,21]. In the mammary gland, dysregulated NLRP3 activation has been repeatedly shown to exacerbate epithelial injury, compromise the blood–milk barrier, and aggravate inflammatory pathology [22,23,24]. Consequently, NLRP3 represents a compelling functional candidate for mastitis resistance, as its regulatory variants may influence both the onset and the severity of intramammary infection. Given that previously identified genetic markers are predominantly associated with pathogen recognition, the inclusion of NLRP3 adds a complementary layer of defense focused on controlling excessive inflammasome-mediated tissue damage. Beyond their utility in marker-assisted selection, polymorphisms in NLRP3 may therefore also serve as predictive indicators of inflammatory severity and therapeutic responsiveness. Future genomic selection strategies in dairy cattle should accordingly consider integrating classical immune-related markers with NLRP3 variants to more effectively reduce mastitis incidence and its associated pathology.

The present review surveys the contemporary literature on the NLRP3 inflammasome in mastitis, with particular emphasis on findings published since 2015. We first consider convergent evidence that positions NLRP3 at the molecular core of mammary inflammation; we then examine pathogen-driven activation, the contribution of oxidative stress and mitochondrial dysfunction, the increasingly recognized gut–mammary and rumen–mammary axes, and the influence of metabolic and thermal stressors. We subsequently appraise the rapidly expanding therapeutic landscape and identify recurring mechanistic motifs before concluding with a synthesis of outstanding questions and priorities for future investigation. Throughout, our objective is not merely to catalog findings, but to articulate the integrative principle that has emerged from them: namely, that NLRP3 functions as a regulatory node at which bacterial, nutritional, metabolic, environmental, and microbial signals converge to shape the clinical phenotype of mastitis.

## 2. Literature Search Methodology

**Figure 1:**
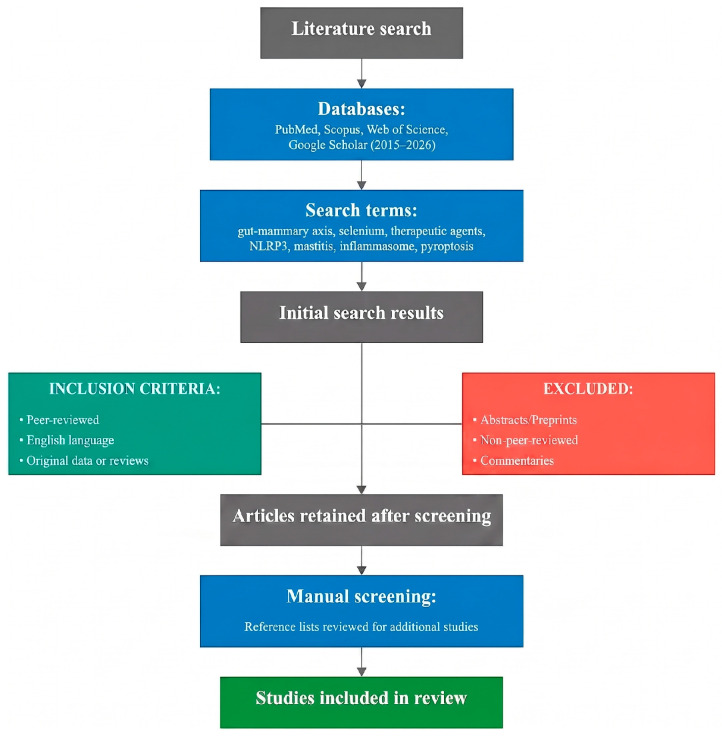
Flow diagram of the literature search and selection process for studies included in this review.

This narrative review was conducted by searching PubMed, Scopus, and Web of Science for articles published between January 2015 and March 2026. The primary search terms included “NLRP3 inflammasome,” “mastitis,” “pyroptosis,” “inflammation and mammary gland,” “gut–mammary axis,” “selenium and inflammasome,” and “therapeutic and NLRP3.” Only peer-reviewed, English-language articles reporting original experimental data or systematic/narrative reviews were considered. Conference abstracts, preprints, and non-peer-reviewed commentaries were excluded. Reference lists of retrieved articles and relevant reviews were manually screened to identify additional studies not captured by the electronic search. As this is a narrative rather than a systematic review, a simple flowchart summarizing the literature identification and selection process has been provided in Figure 1 to enhance transparency of the review process; however, a formal PRISMA flow diagram was not generated. Studies using bovine mammary epithelial cells (bMECs, MAC-T), murine mastitis models, and clinical dairy cow samples were all included, and the model system for each finding is specified throughout the text to enable readers to assess the translational relevance of individual observations. 

## 3. Convergent Evidence for NLRP3 as a Key Hub of Mammary Inflammation

Evidence from multiple independent experimental approaches converges on the NLRP3 inflammasome as a key integrative node in both subclinical and clinical mastitis. Three complementary lines of investigation—proteomic, transcriptomic/cell-based, and regulatory—support this conclusion.

At the proteomic level, analysis of 276 differentially expressed proteins distributed across 17 biological processes identified NLRP3 as a principal node intersecting cytokine production, immune defense, and inflammatory signaling [25]. Pathway enrichment positioned NLRP3, caspase-1 (CASP1), and gasdermin-D (GSDMD) within the NOD-like receptor signaling cascade, and immunohistochemical evaluation confirmed cytoplasmic accumulation with marked upregulation of these proteins at both transcriptional and translational levels in diseased glands [25]. This proteomic evidence suggests that the NLRP3–CASP1–GSDMD axis may play an important role in inflammatory responses during mastitis, potentially representing a defining molecular signature in experimental disease models.

Mechanistic support comes from transcriptomic and cell-based studies. LPS stimulation of bovine mammary epithelial cells produced differential regulation of transcripts and proteins enriched in Toll-like receptor, NF-κB, and NOD-like receptor signaling pathways; NLRP3 depletion subsequently inhibited IL-1β and PTGS2 expression by blocking caspase-1 activity, suggesting NLRP3 as a tractable target in *E. coli* mastitis [26]. Parallel work in MAC-T cells established that *S. aureus* elicits NLRP3 inflammasome activation through K^+^ efflux, triggering ASC recruitment, caspase-1 activation, and GSDMD cleavage, culminating in cellular swelling, membrane rupture, and release of IL-1β and IL-18 [27]. This stepwise dissection brought bovine mastitis into close molecular alignment with the canonical inflammasome paradigm characterized in other systems.

**Figure 2:**
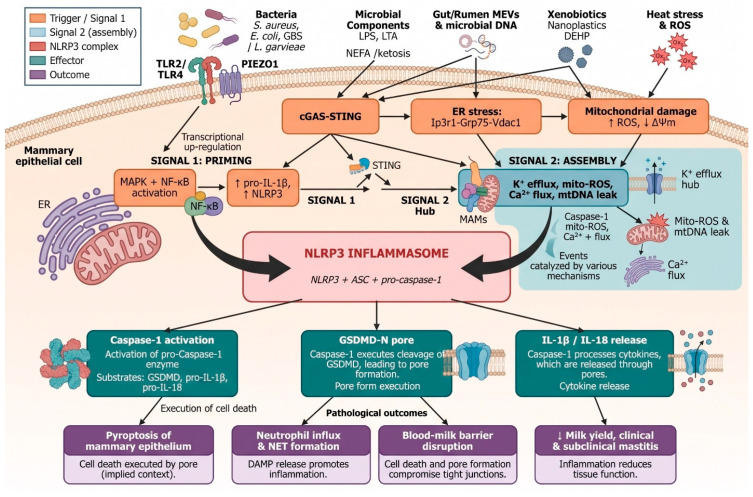
Schematic representation of NLRP3 inflammasome activation in mammary epithelium during mastitis. The figure illustrates the two-signal model: (1) priming via TLR2/TLR4 and NF-κB/MAPK signaling induced by bacterial pathogens, LPS, LTA, non-esterified fatty acids (NEFA), heat stress, environmental xenobiotics, and microbial extracellular vesicles; and (2) activation via ROS, mitochondrial dysfunction, K^+^ efflux, and Ca^2+^ influx that licenses NLRP3 oligomerization with ASC and pro-caspase-1. The downstream consequences—GSDMD pore formation, IL-1β/IL-18 maturation, pyroptosis, and blood–milk barrier disruption—are depicted. Molecular components shown in the figure are listed in table below, along with corresponding references, to avoid textual redundancy.

Beyond pathogen-driven activation, multiple regulatory layers modulate NLRP3 activity in the mammary epithelium. UFL1 negatively regulates NLRP3 inflammasome activation in LPS-stimulated bovine mammary epithelial cells, in part by suppressing NF-κB signaling and ROS production [28]. Consistently, the long non-coding RNA XIST is upregulated in mastitic tissues and generates a negative feedback loop that constrains the NF-κB/NLRP3 pathway [29]. At the post-transcriptional level, bta-miR-223 directly binds to the 3′UTRs of both NLRP3 and Keap1, thereby dampening inflammasome activation and oxidative stress simultaneously [10]. Taken together, these findings indicate that NLRP3 sits at the convergence of transcriptional, post-transcriptional, and proteomic regulation in the mastitic mammary gland, whose activation cascade is summarized in Figure 2.

## 4. Pathogen-Driven Activation of the NLRP3 Axis

Evidence suggests that NLRP3 activation in bovine mastitis may be shaped by a molecular dialog between invading pathogens and innate immune sensors, with the nature of that dialogue varying considerably by pathogen species and virulence strategy in experimental models. *S. aureus* is among the most economically consequential causative agents and has been studied in the greatest mechanistic depth. Its lipoproteins engage TLR2 and TLR4 on macrophages, amplifying inflammatory signaling via the MAPK and NF-κB pathways, with concomitant assembly of the NLRP3 inflammasome. A lipoprotein-deficient *S. aureus* mutant (Δlgt) was substantially less effective than the wild-type strain in inducing IL-1β, IL-6, IL-8, IL-10, and PGE2 production in bovine neutrophils, and pharmacological inhibition of TLR2, TLR4, or NLRP3 each attenuated cytokine and PGE2 output [30]. Bidirectional crosstalk between PGE2 (generated downstream of COX-2 and microsomal prostaglandin E synthase-1) and TLR2, TLR4, and NLRP3 has subsequently been demonstrated, in which pharmacological inhibition of any single component reciprocally attenuates activation of the others [31].

The ultrastructural and molecular consequences of *S. aureus* challenge have been characterized in MAC-T cells, where infection recapitulates the hallmarks of clinical disease: mitochondrial swelling, cristae loss, plasma membrane rupture, and pronounced upregulation of NLRP3, cleaved caspase-1, and the pore-forming N-terminal fragment of gasdermin-D (GSDMD-N) [32]. It is important to note that pyroptosis (caspase-1/GSDMD-mediated inflammatory cell death) and apoptosis (caspase-3/caspase-9-mediated non-inflammatory cell death) represent mechanistically distinct modes of cell death. In the context of mastitis, pyroptosis predominates during acute pathogen challenge and is characterized by membrane pore formation, cellular swelling, and release of pro-inflammatory cytokines, whereas apoptosis occurs under conditions of metabolic stress or as a secondary consequence of severe inflammasome activation. Some pathogens, such as S. aureus, may trigger both pathways depending on bacterial load, virulence factor expression, and the specific cell type affected [20,25].

The pathogen repertoire driving NLRP3 activation extends well beyond *S. aureus*. *Lactococcus garvieae*, a Gram-positive zoonotic pathogen of growing concern in dairy cattle, induces MAC-T cell injury through the TLR2/NLRP3/NF-κB pathway, and NLRP3 silencing attenuates the resulting pyroptosis [33]. *S. agalactiae* mastitis is associated with downregulation of CDK5RAP3, whose loss triggers NF-κB-dependent inflammasome activation, along with accumulation of LC3B and p62—markers of impaired autophagolysosomal degradation—ultimately driving pyroptotic cell death [34]. In bovine mammary epithelial cells stimulated with LPS or *S. agalactiae*, SYK functions as a downstream regulator within a TLR4/SYK/NF-κB axis that constrains NLRP3 expression [35]. A distinct mechanism operates through host mitophagy: PINK1/parkin-mediated mitochondrial clearance dampens NLRP3 inflammasome activation and NF-κB signaling while simultaneously promoting intracellular bacterial persistence, suggesting a pathogen-orchestrated trade-off between acute inflammatory suppression and chronic infection [36]. Across these bacterial models, NLRP3 appears to serve as a key convergence point for diverse microbial provocations, with upstream pathway architecture varying by pathogen while the downstream pyroptotic output remains relatively constant (Table 1).

## 5. Oxidative Stress, Mitochondrial Dysfunction, and the Second Signal

NLRP3 oligomerization requires two sequential signals: a priming event that transcriptionally upregulates inflammasome components, and a second signal that licenses complex assembly. Across the mastitis literature, oxidative stress and mitochondrial dysfunction appear to serve as the second signal in multiple experimental systems. This two-signal paradigm is well established in the broader inflammasome literature, where studies on ASC speck formation [19], NEK7 as an essential mediator of NLRP3 activation, and PKA-mediated phosphorylation of NLRP3 as a regulatory brake have defined the canonical activation mechanism. In the mammary context, these general principles are recapitulated with tissue-specific variations.

Inactivation of *S. aureus* infection of MAC-T cells precipitates a collapse of mitochondrial membrane potential and ROS accumulation, with downstream caspase-1 cleavage and gasdermin-D processing following in sequence. Selenium supplementation (sodium selenite, 2–4 μM), the selective NLRP3 inhibitor MCC950 (10 μM in vitro; 10 mg/kg in murine models via intraperitoneal injection), and the antioxidant N-acetylcysteine (5 mM) each independently abrogate these events, confirming that ROS generation is mechanistically upstream of inflammasome activation, not a passive consequence of it [37]. Consistently, administration of MCC950 (20 mg/kg, intraperitoneally) effectively ameliorated plasma cell mastitis (PCM) by reducing plasma cell infiltration and attenuating pro-inflammatory cytokines, including IL-1β, TNF-α, IL-2, and IL-6, in part by augmenting myeloid-derived suppressor cells (MDSCs) [50]. This finding sits within a broader selenoprotein network encompassing selenoproteins S, O, M, and W, glutathione peroxidase, and thioredoxin reductase, which coordinately regulate redox tone to gate NLRP3 activation. Selenium deficiency aggravates inflammasome assembly through both elevated ROS and altered non-coding RNA expression, while supplementation modulates the TLR/NF-κB/NLRP3, Nrf2/ROS/NLRP3, and TXNIP/NLRP3 axes [51]. The mastitis-specific evidence reinforces this picture: sodium selenite pre-treatment of bovine mammary epithelial cells markedly reduces ROS and suppresses NLRP3, ASC, caspase-1, and IL-1β following *S. aureus* infection [52]; dietary selenium in a murine mastitis model suppresses the same molecular targets [53]; and increasing selenium concentrations progressively attenuate inflammasome expression through combined inhibition of NLRP3 and the NF-κB/MAPK axis [54]. Taken together, these independent lines of evidence suggest that selenium status may be a modifiable determinant of mammary inflammasome susceptibility, particularly in experimental models.

The redox–NLRP3 interface extends to heme oxygenase-1 (HO-1) and thioredoxin-interacting protein (TXNIP). The HO-1 inducer hemin reduces LPS-induced accumulation of mammary ROS, NLRP3 activation, and TXNIP expression, whereas the HO-1 inhibitor ZnPP reverses these effects [55]. A distinct but convergent mechanism operates through endoplasmic reticulum stress and inter-organelle communication. Co-exposure of HC11 mammary epithelial cells to nanoplastics and Di(2-ethylhexyl) phthalate enhances ER–mitochondria crosstalk by upregulating Ip3r1, Grp75, and Vdac1, elevates mitochondrial calcium, and expands membrane contact sites, with these perturbations converging on NLRP3 activation and pyroptotic membrane disruption [43]. This positions environmental xenobiotics as underappreciated triggers of mammary inflammasome activation operating through the same mitochondrial gateway as bacterial pathogens.

## 6. The Gut–Mammary and Rumen–Mammary Axes

Evidence from experimental studies suggests that NLRP3 activation in the mammary gland may be shaped by distal microbial communities, representing a potentially important conceptual framework for understanding mastitis pathogenesis. Rather than a purely local event, mammary inflammasome activity in experimental models appears to reflect the upstream state of gastrointestinal homeostasis—a conclusion supported by mechanistic evidence from multiple independent experimental systems. Importantly, much of the evidence for the gut–mammary axis derives from murine models, and the extent to which these findings are directly translatable to ruminant physiology warrants careful consideration. The bovine gastrointestinal tract differs fundamentally from that of mice in microbial diversity, compartmentalization (rumen, abomasum, intestine), and immune architecture. Where possible, we distinguish between findings obtained in murine and bovine systems, and we highlight the need for validation in dairy cattle throughout this section.

The gut–mammary connection was established causally in a murine model by demonstrating that vagus nerve stimulation attenuates *S. aureus*-induced mastitis through enrichment of Muribaculaceae, with the representative strain S24-7 conferring protection via PPARγ activation and NF-κB/NLRP3 suppression. Fecal microbiota transplantation transferred both the protective microbiota and the protective phenotype, confirming that the effect is microbiota-dependent [56]. Whether vagus nerve–mediated modulation of the gut microbiota operates similarly in ruminants, given the complexity of the ruminant enteric nervous system, remains to be established.

A complementary metabolite-level mechanism operates through the gut tryptophan metabolite indole-3-propionic acid, which attenuates *S. aureus*-induced mastitis by engaging the aryl hydrocarbon receptor, suppressing NF-κB and NLRP3, and restoring blood–milk barrier integrity through upregulation of ZO-1 and occluding [57]. The blood–milk barrier is a selectively permeable epithelial boundary separating the alveolar milk compartment from the interstitial fluid and blood supply of the mammary gland. Its structural integrity depends on apical tight junction proteins—principally claudin-1, claudin-3, occludin, and the cytoplasmic scaffolding protein ZO-1—whose expression and localization are disrupted during both infectious and non-infectious mastitis, leading to increased paracellular permeability, leukocyte infiltration, and elevated somatic cell counts [23,24,45,58].

A parallel rumen–mammary axis has been defined in the context of nutritionally induced mastitis. High-concentrate diet-driven rumen dysbiosis compromises rumen barrier function, promoting translocation of microbial extracellular vesicles carrying bacterial DNA into systemic circulation. These vesicles reach the mammary gland and activate cGAS-STING-NF-κB/NLRP3 signaling; depletion of microbial DNA from the vesicles abrogates their mastitogenic capacity, mechanistically anchoring nucleic acid sensing to NLRP3 activation in non-infectious disease [46]. This distinction between “infectious” mastitis (caused by direct bacterial invasion of the mammary gland via the teat canal) and “non-infectious” or “metabolic” mastitis (driven by translocation of microbial products such as LPS, sialic acid, or extracellular vesicles from the gut or rumen into systemic circulation, which then activate mammary NLRP3 in the absence of intramammary pathogens) is a critical conceptual advance. It implies that a substantial proportion of clinical and subclinical mastitis cases may be amenable to dietary and microbiota-targeted interventions rather than antibiotics.

Transplantation of ruminal microbiota from clinically mastitic cows to mice reproduces mastitis symptoms through the same TLR4-cGAS-STING-NF-κB/NLRP3 cascade, accompanied by mucosal inflammation, impaired intestinal barrier function, and endotoxemia [48]. Bile acid metabolism represents a further metabolic node linking gut dysbiosis to mammary NLRP3 activation. Cows with subacute ruminal acidosis (SARA)-associated mastitis have reduced circulating deoxycholic acid (DCA) levels, and DCA supplementation alleviates *S. aureus*-induced mastitis by activating TGR5, inhibiting NF-κB and NLRP3, and improving blood–milk barrier integrity [24]. Sialic acid metabolism operates through an opposing mechanism: elevated rumen sialic acid in SARA-affected cows promotes expansion of Enterobacteriaceae and Akkermansiaceae, potentiates serum LPS levels, and activates mammary TLR4-NF-κB/NLRP3 signaling. Zanamivir reduced sialic acid levels, dysbiotic taxa abundance, and disease severity, suggesting sialidase inhibition as a microbiota-targeted intervention [47].

**Figure 3:**
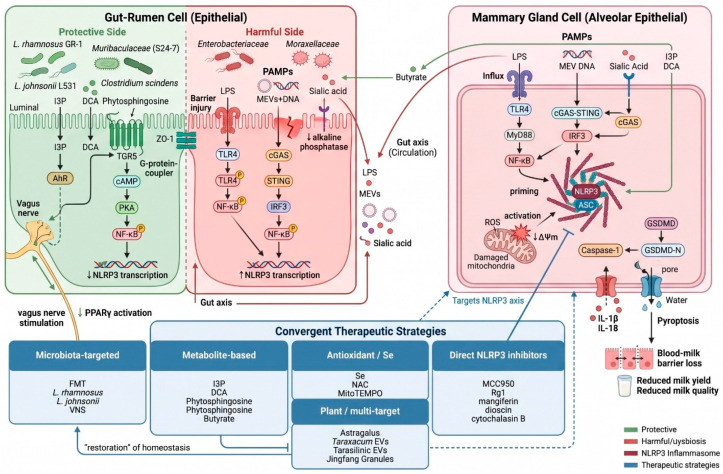
Schematic representation of the gut–rumen–mammary axis and convergent therapeutic targets in mastitis. The figure depicts the bidirectional relationship between gastrointestinal microbial communities and mammary NLRP3 activation. The left panel shows protective microbial taxa and metabolites that suppress NF-κB/NLRP3 signaling; the right panel shows dysbiotic triggers that activate it. Five convergent therapeutic classes are mapped to their respective intervention points along this axis. All molecular interactions shown are supported by references cited in the text; the model system (murine or bovine) for each interaction is indicated to facilitate assessment of translational relevance.

Additional metabolic intersections between the gut and mammary compartments continue to be identified. Phytosphingosine, a sphingolipid metabolite enriched in the rumen and milk of SARA-affected cows, decreases pro-inflammatory cytokines, restores blood–milk barrier function, and inhibits NF-κB and NLRP3 signaling [45]. *S. aureus* infection increases endogenous retrovirus (ERV) transcription and IFN-β levels, and cGAS-STING activation by ERV-derived nucleic acids further drives NF-κB and NLRP3 signaling; emtricitabine-mediated suppression of ERV transcription alleviates mammary injury through this pathway [59]. Thiamine supplementation in SARA-induced goat mastitis simultaneously stabilizes rumen microbiota and circadian rhythm, suppresses NF-κB and NLRP3, and upregulates CLOCK and BMAL1, integrating microbial, immune, and chronobiological control of mammary inflammation within a single experimental model [60]. Collectively, these findings reframe mastitis as a systemic disease in which gastrointestinal homeostasis exerts upstream control over mammary NLRP3 activation, although the clinical relevance of these experimental observations in commercial dairy herds requires prospective validation. The diversity of effective interventions—vagus nerve stimulation, bile acid supplementation, sialidase inhibition, microbiota transplantation—confirms that this axis offers multiple potentially tractable therapeutic entry points, as depicted in Figure 3.

## 7. Metabolic and Hormonal Stress as Modifiers of NLRP3 Activity

Microbial challenge is not a prerequisite for NLRP3 activation in the mammary gland. The transition period in dairy cattle creates conditions of negative energy balance and elevated circulating NEFA that are sufficient to independently drive inflammasome activity. Sinomenine hydrochloride alleviates NEFA-induced injury in bovine mammary epithelial cells by restoring autophagic flux; pharmacological blockade of autophagy with 3-methyladenine abolishes this protection, with downstream consequences including dysregulated antioxidant enzyme activity, elevated expression of NLRP3 and phosphorylated NF-κB, and a shift toward a predominance of pro-inflammatory cytokines [41]. This positions autophagy as a homeostatic brake on inflammasome activation under metabolic stress.

Heat stress operates through a parallel mechanism. Hyperthermia elevates ROS in bovine mammary epithelial cells and activates both NF-κB and NLRP3 signaling; chlorogenic acid mitigates these effects through ROS scavenging, activation of the Nrf2 pathway, and direct inhibition of inflammasome components [42]. A biomechanical dimension is added by the mechanosensitive cation channel PIEZO1, which is upregulated in mammary epithelial cells of clinically mastitic cows and in LPS-induced murine models, and its pharmacological activation with Yoda1 increases NLRP3 expression and apoptosis. Both PIEZO1 silencing and direct NLRP3 inhibition mitigate these effects, defining a PIEZO1–NLRP3 axis through which mechanical and inflammatory signals are integrated in the mammary epithelium [44]. These findings suggest that NLRP3 may function as an integrator of nutritional, thermal, and biomechanical stressors, extending its role beyond that of a microbial sentinel in experimental systems. The implication is that subclinical and clinical masti-tis may arise from inflammasome activation in the absence of intramammary infection, with potential consequences for periparturient disease prevention strategies.

## 8. Therapeutic Targeting of NLRP3 in Mastitis

The therapeutic evidence accumulated in parallel with mechanistic discovery spans five mechanistic classes, summarized in Table 2. To facilitate strategic interpretation, agents are grouped below by their primary mechanistic motif rather than listed sequentially. Three recurring themes emerge across structurally diverse compounds: (i) ROS scavenging and mitochondrial protection, (ii) NF-κB-dependent priming inhibition and autophagy restoration, and (iii) blood–milk barrier preservation. It should be noted that the vast majority of these agents have been evaluated only in cell culture systems or experimental animal models; interventions validated under field conditions in lactating dairy cattle are explicitly indicated where applicable.

### 8.1. ROS Scavenging and Mitochondrial Protection

This mechanistic theme unifies direct NLRP3 antagonists, selenium-based interventions, and several polyphenols that converge on the redox–mitochondrial gateway of inflammasome activation. MCC950, a selective small-molecule NLRP3 inhibitor, provides the most mechanistically transparent tool: it confirms the upstream position of ROS in MAC-T cell inflammasome activation [37], reduces plasma cell infiltration in PCM [50], attenuates apoptosis in ketotic mammary epithelial cells [40], and suppresses NLRP3 activation in *B. cereus*-challenged MAC-T cells [23]. All MCC950 studies to date are preclinical (cell culture or murine models); no field trials in dairy cattle have been reported. The selenium evidence (Section 5) [32,37,51,52,53,54], chlorogenic acid [42], and MitoTEMPO [40] all operate within this same framework. Hemin-mediated HO-1 induction suppresses NLRP3 by reducing TXNIP expression [55]. Dioscin suppresses NLRP3/GSDMD-driven pyroptosis by activating the AMPK/Nrf2 pathway [61]. Cytochalasin B offers a complementary approach by disrupting the ARPC3-, ARPC4-, and HSP70-mediated cytoskeletal rearrangements required for spatial inflammasome assembly in LPS-induced mastitis [22].

### 8.2. Priming Inhibition, Autophagy Restoration, and Microbiota Modulation

The second major therapeutic theme targets the NF-κB-dependent priming step and the autophagic machinery that restrains inflammasome assembly. *Lactobacillus rhamnosus* GR-1 (LGR-1) has the most extensive evidence base: it attenuates ASC-independent NLRP3 activation [62], suppresses both ASC-dependent NLRP3 and NLRC4 inflammasomes [63], induces PINK1/Parkin-mediated mitophagy [38], and protects tight junction integrity [23]. *Lactobacillus johnsonii* L531 similarly inhibits *E. coli*-induced NLRP3 activation and induces ATG5/ATG16L1-mediated autophagy [64]. Among metabolite-level interventions, indole-3-propionic acid [57], deoxycholic acid [24], phytosphingosine [45], sodium butyrate [65], and sodium phenylbutyrate [39] all converge on NF-κB and NLRP3 suppression through distinct receptor-level mechanisms. Maslinic acid achieves modulation of the intestinal flora and direct inhibition of mammary NLRP3, NF-κB, AKT, and MAPK signaling [66].

### 8.3. Blood–Milk Barrier Protection and Multi-Target Plant Compounds

Barrier restoration represents the third convergent therapeutic motif. Morin suppresses NLRP3 inflammasome and NF-κB activation and additionally protects blood–milk barrier integrity by upregulating claudin-3 and occludin via PI3K/AKT, MAPK, and NF-κB inhibition [58,67]. Ginsenoside Rg1 protects the blood–milk barrier in lipoteichoic acid-induced subclinical mastitis by activating PPARγ and regulating AMPK/mTOR signaling to inhibit the ROS/autophagy/NLRP3 axis [68]. Astragalus polysaccharide attenuates LPS-induced mammary fibrosis by suppressing NLRP3, ASC, caspase-1, and IL-1β, and by modulating epithelial–mesenchymal transition markers [69]. *Taraxacum mongolicum*-derived extracellular vesicles simultaneously suppress the NLRP3, NF-κB, and MAPK pathways [70]. The combination of quinic acid and isochlorogenic acid B inhibits NF-κB nuclear translocation, NLRP3 assembly, caspase-11 activation, and gasdermin-D-mediated pyroptosis with efficacies not attainable by either compound alone [71]. Allicin reduces LPS-induced cytokine production and NLRP3 activation via TLR4/NF-κB in MAC-T cells [72]. Jingfang Granules regulate NF-κB, NLRP3, PI3K/AKT, and MAPK pathways while improving tight junction expression [73]. Cytochalasin B provides a complementary approach by disrupting the cytoskeletal rearrangements required for spatial inflammasome assembly [22].

Across this pharmacologically diverse spectrum, the convergence of structurally unrelated compounds on shared molecular nodes reinforces the mechanistic centrality of the NLRP3 axis and supports the rationale for combinatorial regimens targeting multiple steps of the cascade (Table 2). However, it must be emphasized that head-to-head efficacy comparisons, dose-finding studies in lactating cows, and assessments of milk residue profiles are largely absent from the current literature.

**Table 2:** Pharmacological and biological agents targeting the NLRP3 axis in experimental mastitis.

Class	Agent	Primary Mechanism	Model	Evidence Level	Reference
A. ROS scavenging/mitochondrial protection					
Direct NLRP3 inhibitor	MCC950	Selective NLRP3 antagonism; ↓ caspase-1, ↓ GSDMD-N	MAC-T, mouse, PCM	Preclinical (animal)	[23,32,37,50]
Antioxidant/Se	Selenium (Na_2_SeO_3_, diet)	↓ ROS; ↓ NLRP3, ASC, caspase-1	bMECs; mouse	Preclinical (animal)	[52,53,54]
Antioxidant/Se	Selenoprotein F (SELENOF)	Restores ΔΨm; ↓ caspase-1/GSDMD-N	MAC-T	Mechanistic (cell)	[32]
Antioxidant	N-acetylcysteine	ROS scavenging upstream of NLRP3	MAC-T	Mechanistic (cell)	[37,38]
Antioxidant	MitoTEMPO	Mitochondrial ROS scavenging	MAC-T (ketosis)	Mechanistic (cell)	[40]
Antioxidant	Hemin (HO-1 inducer)	↓ TXNIP; ↓ NLRP3	Mouse mammary	Preclinical (animal)	[55]
Polyphenol	Chlorogenic acid	ROS scavenging; Nrf2; ↓ NLRP3	Heat-stressed bMECs	Mechanistic (cell)	[42]
Pyroptosis inhibitor	Dioscin	AMPK/Nrf2 activation; ↓ NLRP3/GSDMD	mMECs; mouse	Preclinical (animal)	[61]
B. Priming inhibition/autophagy/microbiota modulation					
Polyphenol	Morin	↓ NF-κB/NLRP3/MAPK/PI3K-AKT	LPS mouse mastitis	Preclinical (animal)	[58,67]
Polyphenol	Mangiferin	↓ NF-κB; ↓ NLRP3	LPS mouse mastitis	Preclinical (animal)	[74]
Polyphenol combination	Quinic + isochlorogenic acid B	↓ NF-κB; ↓ NLRP3/caspase-11/GSDMD	Mastitis model	Preclinical (animal)	[71]
Probiotic	*L. rhamnosus* GR-1	↓ NLRP3/NLRC4; PINK1/Parkin mitophagy; ↓ ROS	bMECs; MAC-T	Mechanistic (cell)	[23,38,62,63]
Probiotic	*L. johnsonii* L531	↓ NLRP3; ↑ ATG5/ATG16L1 autophagy	Porcine MECs	Mechanistic (cell)	[64]
Insect-derived	*Zophobas morio* hemolymph	↓ NLRP3; ↑ ATG5/ATG16L1 autophagy	*E. coli* mastitis	Preclinical (animal)	[75]
Alkaloid	Sinomenine hydrochloride	Restores autophagic flux; ↓ NLRP3/NF-κB	NEFA-treated bMECs	Mechanistic (cell)	[41]
Microbial metabolite	Indole-3-propionic acid	AhR activation; ↓ NF-κB/NLRP3	Mouse mastitis	Preclinical (animal)	[57]
Microbial metabolite	Deoxycholic acid (DCA)	TGR5 → cAMP/PKA; ↓ NF-κB/NLRP3	S. aureus mouse mastitis	Preclinical (animal)	[24]
Microbial metabolite	Sodium butyrate	↓ NF-κB/NLRP3; histone deacetylase modulation	Bovine macrophages	Mechanistic (cell)	[65]
Microbial metabolite	Sodium phenylbutyrate	↓ TLR2/NF-κB/NLRP3; ↑ defensins	MAC-T (LTA)	Mechanistic (cell)	[39]
Vitamin	Thiamine	↓ NF-κB/NLRP3; ↑ CLOCK/BMAL1	SARA-induced caprine mastitis	Preclinical (animal)	[60]
Antiviral/cGAS-STING	Emtricitabine	↓ ERV transcription; ↓ cGAS-STING-NLRP3	S. aureus mastitis	Preclinical (animal)	[59]
Microbiota intervention	FMT/Clostridium scindens/vagus stimulation	Restores protective taxa; ↓ NF-κB/NLRP3	Mouse/cow	Field-validated	[24,48,56]
Saponin	Ginsenoside Rg1	PPARγ; AMPK/mTOR; ROS/autophagy/NLRP3	LTA subclinical mastitis	Preclinical (animal)	[68]
C. Barrier protection/multi-target					
Pyroptosis inhibitor	Cytochalasin B	Disrupts ARPC3/ARPC4/HSP70 cytoskeletal assembly	LPS-induced mastitis	Preclinical (animal)	[22]
Microbial metabolite	Phytosphingosine	↓ NF-κB/NLRP3; restores tight junctions	*S.aureus* mouse mastitis	Preclinical (animal)	[45]
Plant polysaccharide	Astragalus polysaccharide	↓ NLRP3, ASC, caspase-1; anti-fibrotic	LPS mastitis	Preclinical (animal)	[69]
Plant nanovesicle	Taraxacum mongolicum EVs	↓ NLRP3/NF-κB/MAPK	Mastitis model	Preclinical (animal)	[70]
Organosulfur	Allicin	TLR4/NF-κB; ↓ NLRP3	MAC-T; mouse	Preclinical (animal)	[72]
Triterpenoid	Maslinic acid	↓ NLRP3/AKT-NF-κB/MAPK; gut flora	LPS mouse mastitis	Preclinical (animal)	[66]
TCM formulation	Jingfang Granules	↓ NF-κB/NLRP3/PI3K-AKT/MAPK	LPS mouse mastitis	Preclinical (animal)	[73]
NET inhibitor	Cl-amidine	↓ NETs; ↓ NLRP3/NF-κB/MAPK	LPS mouse mastitis	Preclinical (animal)	[76]
Endogenous regulator	UFL1; bta-miR-223; lncRNA XIST	Negative regulation of NF-κB/NLRP3	bMECs	Mechanistic (cell)	[10,28,29]

Note: We use “↑” to indicate upregulation or increased levels, and “↓” to indicate downregulation or decreased levels.

## 9. Limitations of the Current Evidence Base

The body of evidence reviewed here, while substantial, is subject to several important limitations that qualify the conclusions drawn. A foundational concern pertains to the model systems on which the mechanistic literature relies. The overwhelming majority of studies have been conducted in murine mammary gland models or bovine mammary epithelial cell lines, principally MAC-T cells and primary bMECs. MAC-T cells are an immortalized line derived from bovine mammary alveolar tissue that, while experimentally convenient, may not faithfully recapitulate the heterogeneity of the intact mammary epithelium—particularly with respect to immune cell interactions, hormonal responsiveness, and lactation-stage-dependent phenotypic variation. Primary bMECs provide a closer approximation but are typically isolated from whole mammary tissue without distinction between ductal and alveolar compartments, which may influence the interpretation of NLRP3 activation kinetics and cell death modality. Murine mammary gland models, while enabling genetic manipulation and in vivo assessment, differ from bovine systems in gland architecture, immune cell composition, lactation physiology, and the polymicrobial ecology of naturally occurring mastitis.

Compounding these model-system concerns is the substantial methodological heterogeneity across studies. LPS concentrations range from 1 to 100 μg/mL, treatment durations span 2 to 24 h, and NLRP3 inhibitor dosages (e.g., MCC950: 1–20 μM in vitro; 10–50 mg/kg in vivo) and administration routes (intraperitoneal, intramammary) differ considerably, rendering direct cross-study comparisons difficult. These inconsistencies underscore the need for standardized experimental protocols in mammary inflammasome research.

It should be noted that the vast majority of these agents have been evaluated only in cell culture systems or experimental animal models; clinical interventions validated under field conditions in lactating dairy cattle remain limited and are explicitly indicated where applicable. Furthermore, no NLRP3-targeting therapeutic agent has been evaluated in randomized controlled trials in lactating dairy cows with naturally occurring mastitis. Milk safety data, residue profiles, and withdrawal period assessments—essential prerequisites for any intervention intended for food-producing animals—have not been reported for any of the compounds discussed herein.

An additional translational barrier concerns the absence of standardized biomarkers of mammary inflammasome activity. While NLRP3, caspase-1, and IL-1β are consistently measured across studies, their assessment in milk—rather than tissue lysates—as non-invasive diagnostic indicators have received little systematic attention. The development of milk-based inflammasome biomarker panels would substantially advance both diagnostic stratification and therapeutic monitoring in clinical settings.

Finally, the long-term safety and efficacy of inflammasome-modulating interventions remain entirely uncharacterized. Given the established role of NLRP3 in host defense against intracellular pathogens, sustained pharmacological suppression could theoretically increase susceptibility to secondary infections—a concern that has not been systematically evaluated in any mastitis model to date.

## 10. Synthesis and Future Perspectives

The contemporary literature provides strong experimental support for NLRP3 as a key integrative node through which heterogeneous insults—bacterial, metabolic, dietary, environmental, and thermal—converge to contribute to the clinical phenotype of mastitis. It is nonetheless important to acknowledge that the designation of NLRP3 as “potentially central” rests primarily on experimental models, and the relative contribution of NLRP3 versus other inflammasomes (e.g., NLRC4, AIM2) or inflammasome-independent pathways to naturally occurring bovine mastitis has not been systematically quantified.

Against this backdrop, several priorities for future investigation can be discerned. The relative contributions of epithelial, macrophage, and infiltrating neutrophil NLRP3 activity to overall disease severity remain incompletely defined; cell-type-specific genetic ablation studies will be essential to resolve these contributions with the precision the field now requires.

Combinatorial therapeutic strategies represent an equally pressing avenue for systematic evaluation. Specifically, future studies should assess: (a) dietary selenium supplementation combined with a microbiota-targeted probiotic (e.g., *L. rhamnosus* GR-1), testing whether simultaneous reduction in the oxidative second signal and restoration of gut–mammary axis homeostasis produces additive or synergistic protection; (b) a plant polyphenol (e.g., chlorogenic acid or morin) combined with a direct NLRP3 inhibitor, to determine whether blocking both the priming and activation steps yields greater efficacy than either intervention alone; and (c) bile acid supplementation (DCA) combined with sialidase inhibition (zanamivir), targeting opposing arms of the rumen–mammary metabolic axis simultaneously. These combinations are mechanistically justified by their convergence on distinct steps of the NLRP3 activation cascade and should be evaluated in dose–response studies in lactating dairy cattle under controlled conditions.

The emerging concept that microbial extracellular vesicles bearing nucleic acids can activate NLRP3 [46] invites a broader reconsideration of how subclinical and chronic forms of mastitis are sustained in the absence of overt intramammary infection—an area that has thus far received insufficient mechanistic scrutiny. Of comparable importance is the interplay among autophagy, mitophagy, and inflammasome activity in the lactating mammary gland. PINK1/parkin-mediated mitophagy has been shown to dampen NLRP3 activation while simultaneously promoting intracellular pathogen persistence [36], and this tension warrants dedicated mechanistic investigation in mammary-specific contexts.

Looking further ahead, the integration of selenoproteomics, microbiomics, and metabolomics into unified experimental frameworks holds substantial promise for delineating the upstream determinants of mammary NLRP3 activity at the herd level, and may ultimately provide the mechanistic granularity required to translate experimental insights into durable clinical benefit.

## 11. Conclusions

Mastitis has historically been managed as a localized infectious disease requiring antibiotic intervention, yet the evidence synthesized in this review supports a broader framing. From proteomic signatures of diseased mammary tissue to the mechanistic consequences of gut dysbiosis, rumen acidosis, heat stress, and nanoplastic exposure, findings converge on the NLRP3 inflammasome as a potentially key molecular hub in experimental models of mammary inflammatory pathology. By integrating pathogen- and damage-associated signals, coupling redox tone to cytokine maturation, and executing pyroptotic barrier disruption, NLRP3 may function as a major common effector across etiologically distinct disease forms.

The practical implication is direct: mastitis prevention and therapy cannot be reduced to antimicrobial coverage alone, because a substantial fraction of disease burden may originate from metabolic, nutritional, and environmental inputs that antibiotics cannot address. Selenium status, rumen microbiota composition, gut barrier integrity, bile acid metabolism, and circadian regulation are now identified as candidate upstream determinants of mammary inflammasome susceptibility, each of which is amenable to dietary or managerial adjustment without recourse to antibiotics.

Translation to practice will require moving beyond single-agent, single-pathogen experimental designs. Most studies reviewed here relied on murine LPS or mono-species infection models that, while mechanistically illuminating, do not capture the polymicrobial complexity, metabolic co-morbidities, or production pressures of commercial dairy herds. Validation of NLRP3-targeting strategies in lactating cows under field conditions—with attention to milk safety, residue profiles, and herd-level efficacy—represents the most urgent translational gap.

## Figures and Tables

**Table 1:** Upstream triggers and signaling pathways converging on NLRP3 activation in mastitis.

Trigger/Stimulus	Model Origin	Upstream Signaling Pathway	Inflammasome Readout	Reference
*Bacillus cereus* 2101	Cell culture (MAC-T)	NLRP3/caspase-1/GSDMD	Loss of ZO-1/occludin; cell death	[23]
*S. aureus* lipoprotein	Primary bovine (neutrophils)	TLR2/TLR4/NLRP3 → MAPK, caspase-1	↑ IL-1β, IL-6, IL-8, PGE2	[30]
PGE2/COX-2/mPGES-1	Bovine clinical (*S. aureus* mastitis)	Bidirectional TLR2/TLR4–NLRP3	Mutual reinforcement of inflammasome priming	[31]
*L. garvieae* LG41	Cell culture (MAC-T) + murine	TLR2/NLRP3/NF-κB	Pyroptosis; rescued by NLRP3 silencing	[33]
*S. agalactiae*	Primary bovine + cell culture (bMECs)	CDK5RAP3 loss → NF-κB/NLRP3	Caspase-1 cleavage, pyroptosis	[34]
*S. agalactiae* (GBS)	Cell culture (bMECs)	TLR4/SYK/NF-κB/NLRP3	↑ IL-1β, IL-8, NLRP3	[35]
*S. aureus* (live and inactivated)	Cell culture (MAC-T)	Mitochondrial ROS → NLRP3	↑ NLRP3, cleaved caspase-1, GSDMD-N	[32,37]
*E. coli*	Cell culture (MAC-T) + murine	ROS-dependent NLRP3	NLRP3, caspase-1, apoptosis	[26,38]
LPS/LTA	Cell culture (bMECs) + murine	TLR4/NF-κB/NLRP3	↑ IL-1β, IL-18; pyroptosis	[26,39]
NEFA (ketosis)	Cell culture (MAC-T) + primary bovine	Mito-ROS → NLRP3	Mitochondrial damage, apoptosis	[40,41]
Heat stress (hyperthermia)	Cell culture (bMECs)	ROS/NF-κB/NLRP3	↑ IL-1β, IL-6, pyroptosis	[42]
Nanoplastics + DEHP	Cell culture (HC11, murine)	ER–mitochondria contact (Ip3r1/Grp75/Vdac1) → NLRP3	Pyroptosis, ↑ Ca^2+^, ↓ ΔΨm	[43]
PIEZO1 activation (Yoda1)	Cell culture (MAC-T) + murine	PIEZO1 → NLRP3	Apoptosis, ↑ NLRP3	[44]
Endogenous retroviruses (ERVs)	Bovine/murine mammary (infected)	cGAS-STING → NF-κB/NLRP3	Reversed by emtricitabine	[45]
Microbial extracellular vesicles	Murine (rumen→mammary)	cGAS-STING-NF-κB/NLRP3	Non-infectious mastitis	[46]
Sialic acid (from SARA rumen)	Murine model	TLR4-NF-κB/NLRP3	Gut + mammary inflammation	[47]
Recurrent low-grade LPS	Murine model	TLR4-cGAS-STING-NF-κB/NLRP3	Severe mastitis	[48]
NET-derived histones	Cell culture (bMECs)	Caspase-1/3 + NLRP3	Necrosis, pyroptosis, apoptosis	[49]

Note: We use “↑” to indicate upregulation or increased levels, and “↓” to indicate downregulation or decreased levels.

## Data Availability

No new data were created or analyzed in this study.
